# Drought tolerance of cowpea is associated with rapid abscisic acid biosynthesis via VuWRKY57 in root

**DOI:** 10.1093/aobpla/plaf038

**Published:** 2025-07-08

**Authors:** Misaki Tochihara-Tanaka, Sayuri Kai, Nao Murakami, Shinya Murakami, Nene Miura, Reika Komai, Yuka Tatsumi, Mio Takahashi, Norimitsu Hamaoka, Mari Iwaya-Inoue, Chetphilin Suriyasak, Yushi Ishibashi

**Affiliations:** Faculty of Agriculture, Kyushu University, 744 Motooka, Nishi-ku, Fukuoka 819-0365, Japan; Faculty of Agriculture, Kyushu University, 744 Motooka, Nishi-ku, Fukuoka 819-0365, Japan; Faculty of Agriculture, Kyushu University, 744 Motooka, Nishi-ku, Fukuoka 819-0365, Japan; Faculty of Agriculture, Kyushu University, 744 Motooka, Nishi-ku, Fukuoka 819-0365, Japan; Faculty of Agriculture, Kyushu University, 744 Motooka, Nishi-ku, Fukuoka 819-0365, Japan; Faculty of Agriculture, Kyushu University, 744 Motooka, Nishi-ku, Fukuoka 819-0365, Japan; Faculty of Agriculture, Kyushu University, 744 Motooka, Nishi-ku, Fukuoka 819-0365, Japan; Faculty of Agriculture, Kyushu University, 744 Motooka, Nishi-ku, Fukuoka 819-0365, Japan; Faculty of Agriculture, Kyushu University, 744 Motooka, Nishi-ku, Fukuoka 819-0365, Japan; Faculty of Agriculture, Kyushu University, 744 Motooka, Nishi-ku, Fukuoka 819-0365, Japan; Faculty of Agriculture, Kyushu University, 744 Motooka, Nishi-ku, Fukuoka 819-0365, Japan; Faculty of Agriculture, Kyushu University, 744 Motooka, Nishi-ku, Fukuoka 819-0365, Japan; Phenome, Genome & Environment

**Keywords:** abscisic acid, cowpea, drought stress, WRKY

## Abstract

Cowpea [*Vigna unguiculata* (L.) Walp] is more drought tolerant than other legumes, although drought still limits its productivity. Under drought stress, cowpea closes its stomata more rapidly to maintain its plant water content than soybean. The rapidly stomatal closure under drought stress in cowpea is mediated by abscisic acid (ABA), but the details of the mechanism are not yet clear. We examined the expression of an ABA-biosynthesis-related gene encoding 9-*cis*-epoxycarotenoid dioxygenase (NCED) and ABA content in roots of cowpea and soybean under drought stress. Following an analysis of each *NCED* gene’s promoter, we investigated the expression of the genes for WRKY transcription factors VuWRKY57 and GmWRKY32. *NCED* gene expression conformed with *VuWRKY57* and *GmWRKY32* expression, and VuWRKY57 bound to the *VuNCED1* promoter. Overexpression of *VuWRKY57* in *Arabidopsis* confirmed the drought stress tolerance. Thus, under drought stress, *VuWRKY57* expression is rapidly induced in cowpea roots; ABA content increased via the induction of *VuNCED1* leads to stomatal closure; and drought tolerance is conferred.

## Teaser

Cowpea is known for its strong drought tolerance compared to other crops. One reason is its ability to quickly close stomata-tiny openings on leaves-to conserve water during dry conditions. This response is triggered by a plant hormone called abscisic acid (ABA). In this study, it found that a gene named VuWRKY57 becomes highly active in cowpea roots under drought stress and stimulates the production of ABA by activating another gene, VuNCED1. This increase in ABA helps the plant close stomata faster and tolerate drought better. When VuWRKY57 was introduced into Arabidopsis, it also improved drought resistance, suggesting it may be useful for developing drought-resilient crops.

## Introduction

Drought stress in plants induces stomatal closure, represses growth and photosynthesis, and activates respiration. It is the primary cause of crop loss worldwide, reducing average yields of most major crop plants (Boyer 1982; Bray et al. 2000; Zhu 2002; Nakagawa et al. 2018). The cost of drought damage around the world is estimated at USD 307 billion per year (Mirzabaev et al. 2024). Drought is the most serious of the environmental stresses that limit global crop production; for example, drought reduces yields of soybean [*Glycine max* (L.) Merr.] by 46%–71% (Daryanto et al. 2017; Fahad et al. 2017).

Cowpea (*Vigna unguiculata*) is an important legume crop that provides a source of protein-rich food. Africa is responsible for >90% of world production (FAOSTAT 2024), with the drier Savannah and the Sahelian region of West and Central Africa being responsible for producing 72% of the total. Although cowpea is recognized as more drought tolerant than other crops including lablab beans, bambara groundnuts, peanuts, pearl millet, sorghum, green gram, dark gram, maize, and soybeans (Hall 2012), drought is still a major constraint on its productivity in Africa (Nkomo et al. 2021). Under drought stress, its stomata close rapidly, reducing transpiration (Hall and Schulze 1980; Imamura et al. 2010; Belko et al. 2013). Many cowpea genes involved in drought responses are recognized (Carvalho et al. 2017). Using a differential screening method, Iuchi et al. (1996a) isolated 24 cDNA clones that corresponded to 10 dehydration-induced genes from a highly drought-tolerant cowpea cultivar, nine of which were specific to dehydration stress. Iuchi et al. (1996a, b) characterized five of them (*cowpea responsive to dehydration*: *CPRD8*, *CPRD14*, *CPRD22*, *CPRD12*, and *CPRD46*) and described two additional drought-inducible genes from the same cultivar, *VuNCED1* and *VuABA1*. *VuNCED1* encodes a 9-*cis*-epoxycarotenoid dioxygenase that catalyses a key step in abscisic acid (ABA) biosynthesis, and *VuABA1* encodes a zeaxanthin epoxidase involved in another step of ABA biosynthesis (Iuchi et al. 2000), and which is required for resistance to osmotic and drought stress, ABA-dependent stomatal closure, and regulation of the expression of stress-responsive genes (Seo and Koshiba 2002).

ABA regulates not only many essential processes of plant development, including germination, but also several adaptive responses to a variety of environmental stresses; in particular, it is one of the main regulators of plant drought tolerance (Finkelstein et al. 2002; Fujita et al. 2005). Studying the response of 6 cowpea cultivars to drought stress, Kulkarni et al. (2000) suggested that the intrinsic capacity for ABA synthesis could play an important role in regulating stomatal conductance; notably, drought-stressed plants accumulate more ABA than unstressed plants (Agbicodo et al. 2009). In cowpea, *VuNCED1* has a key role in the biosynthesis of ABA under drought stress (Iuchi et al. 2000).

WRKY transcription factors play important roles in biotic and abiotic stress responses (Eulgem and Somssich 2007; Miller et al. 2008). Many *WRKY* genes are involved in the responses to various sources of abiotic stress (Miller et al. 2008; Chen et al. 2012). In wheat, 8 of 15 *WRKY* genes were transcribed in response to cold, heat, NaCl, and PEG treatment (Wu et al. 2008). In the roots of Arabidopsis plants treated with 150 mM NaCl, 18 AtWRKY genes were induced (Jiang and Deyholos 2006). Overexpression of stress-induced *OsWRKY45* enhanced drought tolerance in rice and *Arabidopsis* (Qiu and Yu 2008; Tao et al. 2011). Additionally, overexpression of *OsWRKY30* activated by a MAPK cascade also enhanced the tolerance of the transgenic rice to drought (Li et al. 2012).

WRKY proteins and ABA influence stress-induced responses. WRKY20 from Glycine soja regulates ABA signalling and enhanced drought tolerance in *Arabidopsis* (Luo et al. 2013). WRKY63 in Arabidopsis enhances drought tolerance: disruption of *At*WRKY63/ABA Overly Sensitive3 (*ABO3*) increased ABA sensitivity and reduced drought tolerance, the latter in large part by blocking responsiveness of stomatal closure to ABA (Banerjee and Roychoudhury 2015). AtWRKY63 binds to the promoters of ABA Responsive Element Binding Proteins/Factors (*AREB1/ABF2*) (Li 2014). Overexpression of *AtWRKY57* in *Arabidopsis* improved drought tolerance by directly targeting the *AtNCED3* promoter and promoting ABA biosynthesis (Jiang et al. 2012). Additionally, overexpression of the *McWRKY57-like* gene from *Mentha canadensis* L. may improve water uptake, maintain chlorophyll content stability, and reduce water loss under water-deficit conditions, thus enhancing plant drought tolerance (Bai et al. 2023). This evidence prompts our hypothesis that drought tolerance of cowpea might be improved through manipulation approaches of the WRKY57. Here, therefore, we examined the ABA biosynthesis through the VuWRKY57 and VuNCED1 and clarify the role of VuWRKY57 under drought stress in cowpea.

## Materials and methods

### Plant materials

Cowpea [*Vigna unguiculata* (L.) Walp. cv. IT98K-205-8, drought-tolerant line (Ishikawa et al. 2019)], and soybean [*Glycine max* (L.) cv. Fukuyutaka, drought-sensitive cultivar (Ishibashi et al. 2011)], were sown in 1.4-L pots filled with sand in a greenhouse at Kyushu University, Japan. Basal fertilizer (N:P:K = 3:10:10) and magnesium lime were applied at each 1.25 g pot^−1^. After emergence, seedlings were thinned to one plant per pot. Plants were grown until the appearance of the third leaf, and then irrigation was stopped. In the control treatment, plants were irrigated until soil saturation once a day throughout the experiment. The pots were arranged in a completely randomized design. All measurements consisted of five replicates (five plants per treatment).

### Soil water content

At the completion of each treatment period, the soil in each pot in cowpea, soybean, and *Arabidopsis* was divided into 0- to 10-cm depth and that below, weighed immediately, and then dried for 48 h at 90°C. The soil water content was calculated as [(wet weight)−(dry weight)]/(wet weight).

### Relative water content in leaf and root

To measure the relative water content (RWC) in cowpea and soybean, the first leaf (next of primary leaf) and whole root were sampled daily after start drought treatment. The RWC was determined as [(fresh weight−dry weight)/(turgid weight−dry weight)]. Water content was determined by oven-drying at 80°C for 48 h, and full turgor was estimated by keeping the samples overnight in water at 4°C.

### Stomatal conductance

Stomatal conductance of the first leaf during drought stress was measured with a leaf porometer (SC-1; Decagon Devices, Pullman, WA, USA) in the morning (08:00–11:00) and recorded 2 min after sensor head had been clipped to the leaf.

### Real-time PCR analysis

Total RNA was extracted from leaves and roots in cowpea, soybean, and *Arabidopsis* during drought stress by the SDS/phenol/LiCl method (Chirgwin et al. 1979). cDNA was synthesized and amplified as described by Ishibashi et al. (2017) using ReverTra Ace reverse transcriptase (Toyobo). The amount of each gene transcript was normalized against the amount of *VuActin* mRNA by the method of Pfaffl (2001). The *VuActin* primer sequences came from Tatsumi et al. (2019); the other primer sequences are given in Table 1.

**Table 1.** Primers used in this study.

Gene name	Accession No.	Sequence
Vector construction		
35S F		CTATCCTTCGCAAGACCCTTC
VuWRKY57 entry F		CACCATGGAAGACAAGGACAGA
VuWRKY57 stop R		TCATCTGGTACGCATTCCCGGA
Subcellular localization		
GFP F		TCAAGATCCGCCACAACATCGA
VuWRKY57 entry F		CACCATGGAAGACAAGGACAGA
VuWRKY57 stop R		TCATCTGGTACGCATTCCCGGA
EMSA		
pGEX4T-1 F		GGACCCAATGTGCCTGGATGCG
VuWRKY57 F		GGTTCCGCGTGGATCCCCGGAATTCATGGAAGACAAGGACAGAG
VuWRKY57 R		GTCAGTCACGATGCGGCCGCTCGAGTCATCTGGTACGCATTCC
VuNCED1 W-box1 F		TGTGGGGCAATTGGGATGTGACGTGGGGA
VuNCED1 W-box1 R		TCCCCACGTCACATCCCAATTGCCCCACA
VuNCED1 W-box2 F		CCACTGAGTCACCCAATCCACACTAAAAA
VuNCED1 W-box2 R		TTTTTAGTGTGGATTGGGTGACTCAGTGG
VuNCED1 W-box3 F		TACATGTGTCACTTCAACCTCCATCCTCT
VuNCED1 W-box3 R		AGAGGATGGAGGTTGAAGTGACACATGTA
qRT-PCR		
VuEF1b-pRT_F	AB588747	GAAGACAAAAAGGCAGCAGAGGAA
VuEF1b-pRT_R		CCTGATGTGCTGACTACTACTGCAT
VuNCED1-qRT_F	AB030293	AAGCATGCCTTCATCAGCTT
VuNCED1-qRT_R		GGGGAAGTGGAGTGTTTGAA
VuWRKY57-qRT_F	FG882047	GGCTACAGATGGCGGAAGTA
VuWRKY57-qRT_R		TCTTCTTCACCGTGCATTTG
GmEF1b-qRT_F	NM_001249608	GAAGACAAAAAGGCAGCAGAGGAA
GmEF1b-qRT_R		TCCACAGATACAAGGTCATCGACA
GmNCED3-qRT_F	XR_418624	CGAATGCGATGAGAGTTTGA
GmNCED3-qRT_R		TTTCGTCCGAGCTTGTTTCT
GmWRKY32-qRT_F	XM_006573077	GATGAAGGGTTGCTTGGAGA
GmWRKY32-qRT_R		CACCTCCTATATATTATCCGAGATTC
AtActin2-qRT_F	NM_001338359	AAGCTGGGGTTTTATGAATGG
AtActin2-qRT_R		TTGTCACACACAAGTGCATCAT
AtWRKY57-qRT_F	NM_001334406	TTCACAGAGACAATAATGCTCCTTC
AtWRKY57-qRT_R		TCTGATGGAGTAGACACGGCAG
AtNCED3-qRT_F	NM_112304	CCAGATTGCTTCTGCTTCCAT
AtNCED3-qRT_R		GACCCTATCACGACGACTTCATC
AtP5CS-qRT_F	NM_001202785	GGTGGACCAAGGGCAAGTAAGATA
AtP5CS-qRT_R		TCGGAAACCATCTGAGAATCTTGT
VuWRKY57-arabidopsis-qRT_F	FG882047	CAGCCGGAGAAATCCACAGT
VuWRKY57-arabidopsis-qRT_R		TAAATGCAAACCGTGGCTGC
RT-PCR (transgenic)		
VuWRKY57-RT_F	FG882047	CACCATGGAAGACAAGGACAGA
VuWRKY57-RT_R		TCATCTGGTACGCATTCCCGGA
VuActin-RT_F	XM_028073553	GCGTGATCTCACTGATGCCCTTAT
VuActin-RT_R		AGCCTTCGCAATCCACATCTGTTG

### Abscisic acid content

To measure the ABA content in cowpea and soybean, the roots at 4 days after drought treatment were sampled and stored them at −80°C. Their ABA levels were measured with a Phytodetek Competitive ELISA kit (Agdia, Elkhart, IN, USA). Each experiment used 5 biological replicates of each treatment.

### Particle bombardment

For particle bombardment, 1 µg of reporter plasmid or internal control plasmid was precipitated onto 0.6-mg gold particles (1.0 μm diameter). Plasmids with the *35S:GFP-VuWRKY57* fusion in the pGWB506 Gateway bSinary vector (Nakagawa et al. 2007) were bombarded into onion epidermal cells at 6.2 MPa from a PDS1000 He Biolistic Particle Delivery System (Bio-Rad; Hercules, CA, USA). The tissues were then kept on 1/2 MS medium plates for 12 h at 27°C in darkness. The fluorescence signal of the GFP-VuWRKY57 fusion protein was imaged under a fluorescence microscope (Keyence; BZ-710). The 35S:GFP vector was used as a control.

### Electrophoretic mobility shift assay

Electrophoretic mobility shift assays (EMSA) were performed as described by Zhou et al. (2018). Recombinant GST-VuWRKY57 protein was produced in *Escherichia coli* BL21 (DE3) pLysE cells. The cells were lysed by sonication and the protein was purified with glutathione–Sepharose 4B beads (GE Healthcare). A double-stranded oligonucleotide spanning the TGAC core motif upstream of *VuNCED1* (Vu01g052600) was prepared and was labelled as probe according to the protocol provided with a DIG Kit (Roche Diagnostics). Assays were carried out using a DIG Gel Shift kit, 2nd Generation (Roche Diagnostics), according to the manufacturer’s instructions.

### Generation of *Arabidopsis* transgenic lines

To generate the *35S:VuWRKY57* construct, we integrated a cDNA fragment containing the full VuWRKY57 coding sequence (*Vu02g051100*) from the cloning plasmid into the pGWB502 Gateway binary vector (Nakagawa et al. 2007) in sense orientation behind the CaMV 35S promoter. *Arabidopsis* plants (Col-0) were transformed by the floral dip procedure (Clough and Bent 1998). Seeds were collected from the transformed plants and selected on 1/2 MS medium supplemented with hygromycin. Hygromycin-resistant plants were transferred to soil 14 days after germination and were grown in a growth chamber. Wild type (Col-0) and T_3_ Arabidopsis seeds, seedlings and plants were grown in a controlled environment cabinet at 22°C in 16/8-h light/dark regime. *Arabidopsis* seeds were germinated on a MS medium. Each 10 two-week-seedlings were transplanted to the same pot. At 14 days after transplanting, irrigation was stopped. Col-0 and T_3_ plants were conﬁrmed the expression of *VuWRKY57* and *AtNCED3* by quantitative real-time PCR analysis and were analysed false-colored infrared-thermal images (FLIR C2; FLIR Systems AB, Wilsonville, OR, USA). To analyse leaf water status, the detached leaves grown under control conditions were weighed at 30 min intervals for up to 5 h as described by Quan et al. (2016). When the irrigation was stopped for 35 days, those plants were measured for their survival rate.

### Statistical analysis software

The experiments were performed in a completely randomized design. All measurements were performed using a simple random sampling method. Statistical analysis was performed in Statcel4 software (OMS Publishing Inc., Tokyo, Japan). Differences among treatments were analysed by performing a one-tailed Student’s *t*-test and Tukey’s test.

## Results

### Physiological characteristics of cowpea and soybean under drought stress

**Figure 1.**
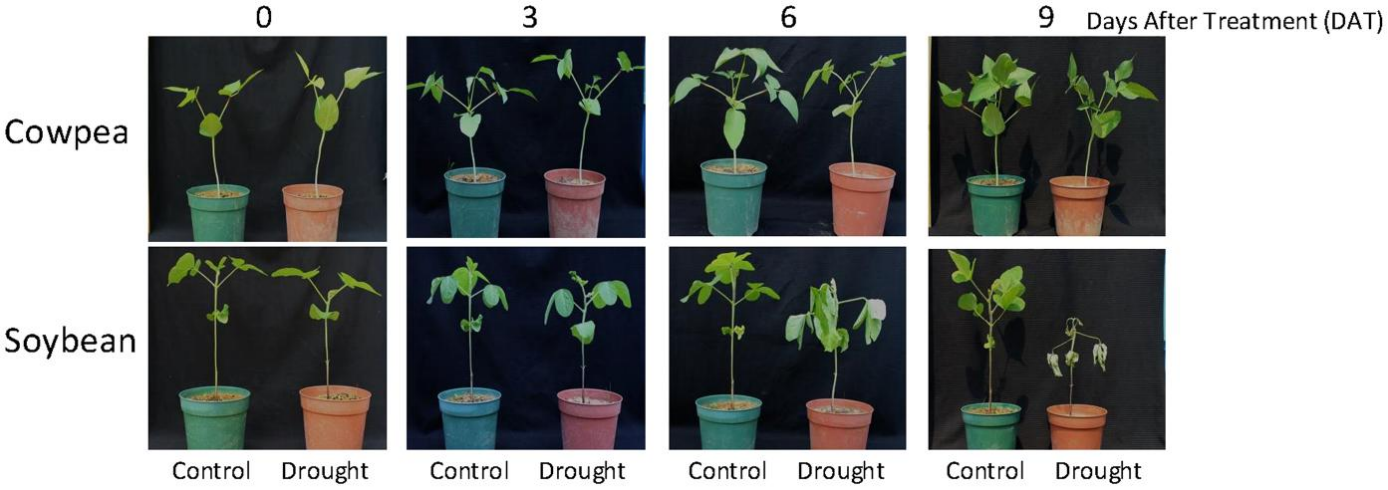
Phenotypes of cowpea and soybean under drought stress.

**Figure 2.**
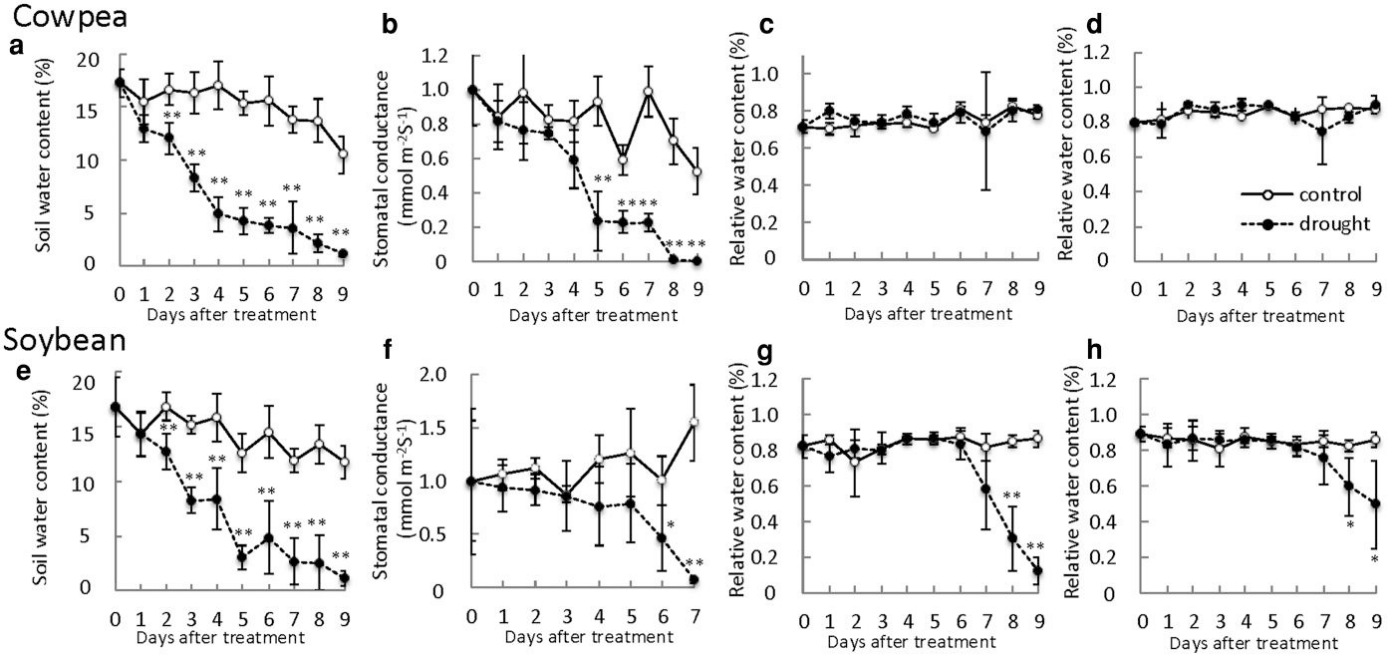
Physiological responses of cowpea and soybean under drought stress. (a-d) cowpea. (e-h) soybean. (a, e) Soil water content. (b, f) Stomatal conductance. (c, g) Relative water content in leaf. (d, h) Relative water content in root. White and black circles mean control and drought stress, respectively. Asterisks indicate significant differences (**P < 0.01, *P < 0.05, Student’s *t*-test). Values are mean ± S.D. (*n* = 5).

During the 9 days when irrigation was withheld (Fig. 1), the soil water content gradually decreased (Fig. 2a and e). The stomatal conductance of cowpea decreased from 1 day after the irrigation was stopped and was significantly lower from 5 days than in the control (Fig. 2b). That of soybean decreased from 6 days (Fig. 2f). The relative water contents of leaf and root in cowpea did not differ significantly between treated and control plants during treatment (Fig. 2c and d), but those in soybean were significantly less than in the control from 8 days (Fig. 2g and h). These results show that cowpea retained water better than soybean.

### Expression of abscisic acid biosynthesis-related genes and abscisic acid content in cowpea and soybean under drought stress

**Figure 3.**
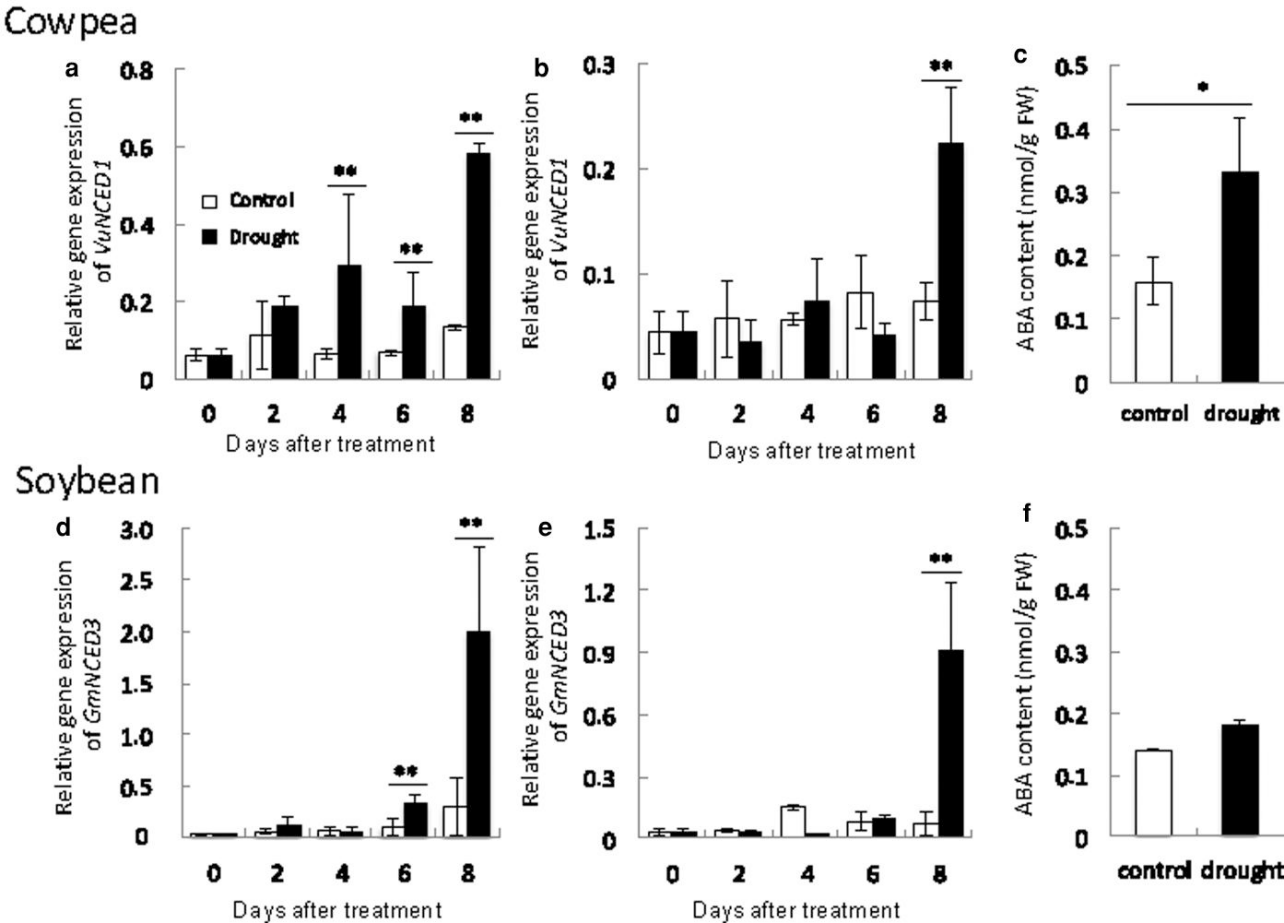
Expression of *NCED* genes and ABA contents in cowpea and soybean under drought stress. (a-c) cowpea. (d-f) soybean. *VuNCED1* expressions in roots (a) and leaves (b) of cowpea. *GmNCED3* expressions in roots (d) and leaves (e) of soybean. ABA content in roots of at 4 days of drought stress in cowpea (c) and soybean (f). White and black bars mean control and drought stress, respectively. Asterisks indicate significant differences (**P < 0.01, *P < 0.05, Student’s *t*-test). Values are mean ± S.D. (*n* = 5).

In cowpea under drought stress, the expression of *VuNCED1*, ABA biosynthesis–related gene, is one of the key genes for the immediately stomatal closure (Iuchi et al. 2000). In this study, under drought stress, the expression of *VuNCED1* in cowpea roots gradually increased and was significantly higher than in the control from 4 days (Fig. 3a). The expression of *GmNCED3*, which is gene orthologous to *VuNCED1*, in soybean roots was significantly higher than in the control from 6 days (Fig. 3d). Those in leaves of both species were significantly higher at 8 days (Fig. 3b and e). These results indicate that the expression of *NCED* genes in roots explains the difference between cowpea and soybean. Therefore, we investigated the ABA content of root in both plants. The ABA content at 4 days was significantly higher than in the control in cowpea roots but was unchanged in soybean roots (Fig. 3c and f). Eventually, ABA contents at 8 days after drought stress treatment in both plants increased in root (Supplementary Figure S1). Together, these data suggested that the immediately stomatal closure in cowpea under drought stress depends on ABA biosynthesis through the rapid expression of *VuNCED1* in root.

### *WRKY* expression in cowpea and soybean under drought stress

**Figure 4.**
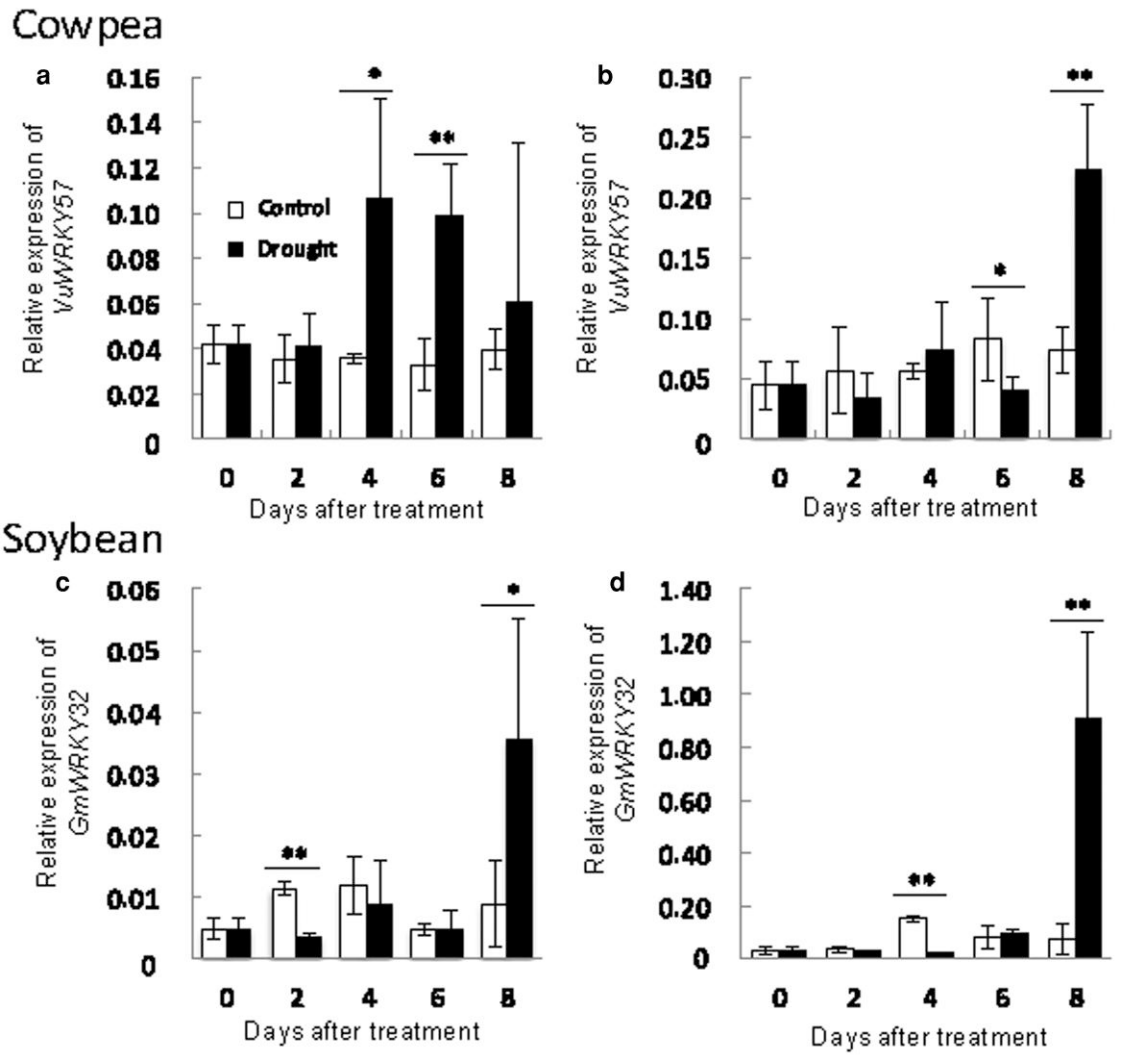
Expression of *WRKY* genes in cowpea and soybean under drought stress. (a, b) cowpea. (c, d) soybean. *VuWRKY57* expressions in roots (a) and leaves (b) of cowpea. *GmWRKY32* expressions in roots (c) and leaves (d) of soybean. White and black bars mean control and drought stress, respectively. Asterisks indicate significant differences (**P < .01, *P < .05, Student’s *t*-test). Values are mean ± SD (*n* = 5).

In *Arabidopsis*, AtWRKY57 was binding to the promoter of *AtNCED3* and consequently promoted the ABA biosynthesis (Jiang et al. 2012). We, therefore, analysed the homologue of AtWRKY57 in cowpea and soybean, *VuWRKY57* and *GmWRKY32* (Fig. 4). The expression of *VuWRKY57* in cowpea roots was significantly higher under drought stress than in the control from 4 days (Fig. 4a), but that of *GmWRKY32* in soybean roots was rapidly higher at 8 days (Fig. 4c). The expression of both genes in leaves was rapidly higher at 8 days (Fig. 4b and d). Interestingly, the expression of WRKYs synchronized to the expression of NCEDs in both plants.

### Function of VuWRKY1

**Figure 5.**
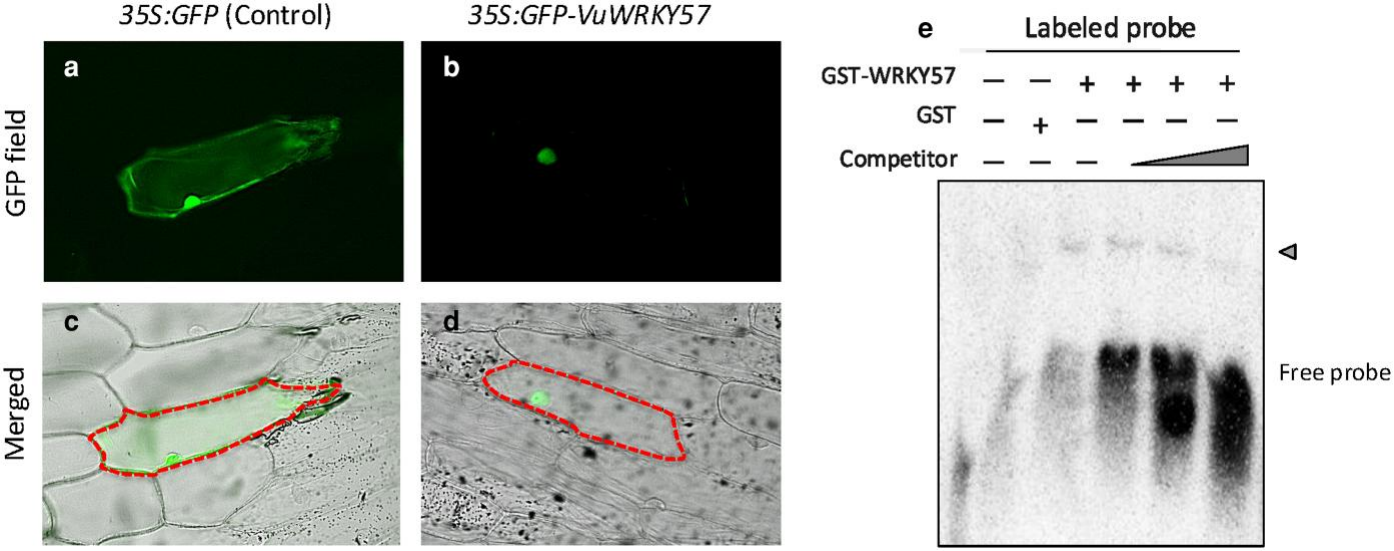
Cellular localization of VuWRKY57 in onion epidermal cells and its binding to *VuNCED1* promoter. (a, c) 35S:GFP (control). (b, d) 35S:GFP-VuWRKY57. (e) EMSA, The retarded DNA–protein complex was reduced by competition with either labelled or unlabelled (competitor) probe at a 20×, 50×, and 100× molar excess (3 right-hand lanes). Arrowhead indicates position of shifted bands.

VuWRKY57 was localized in onion cell nuclei (Fig. 5b and d). As drought stress synchronously induced the expression of *VuNCED1* and *VuWRKY57* (Figs. 2a and 3a), we confirmed the *VuNCED1* promoter sequence and found the W-box, which is a *cis*-acting element recognized by several WRKY transcription factors (Banerjee and Roychoudhury 2015). To test this possibility, we, therefore, used EMSA to investigate whether VuWRKY57 binds directly to the *VuNCED1* promoter containing the W-box. The GST-VuWRKY57 recombinant protein bound to the *VuNCED1* promoter fragment, and DNA binding was suppressed by a competitor (Fig. 5e).

**Figure 6.**
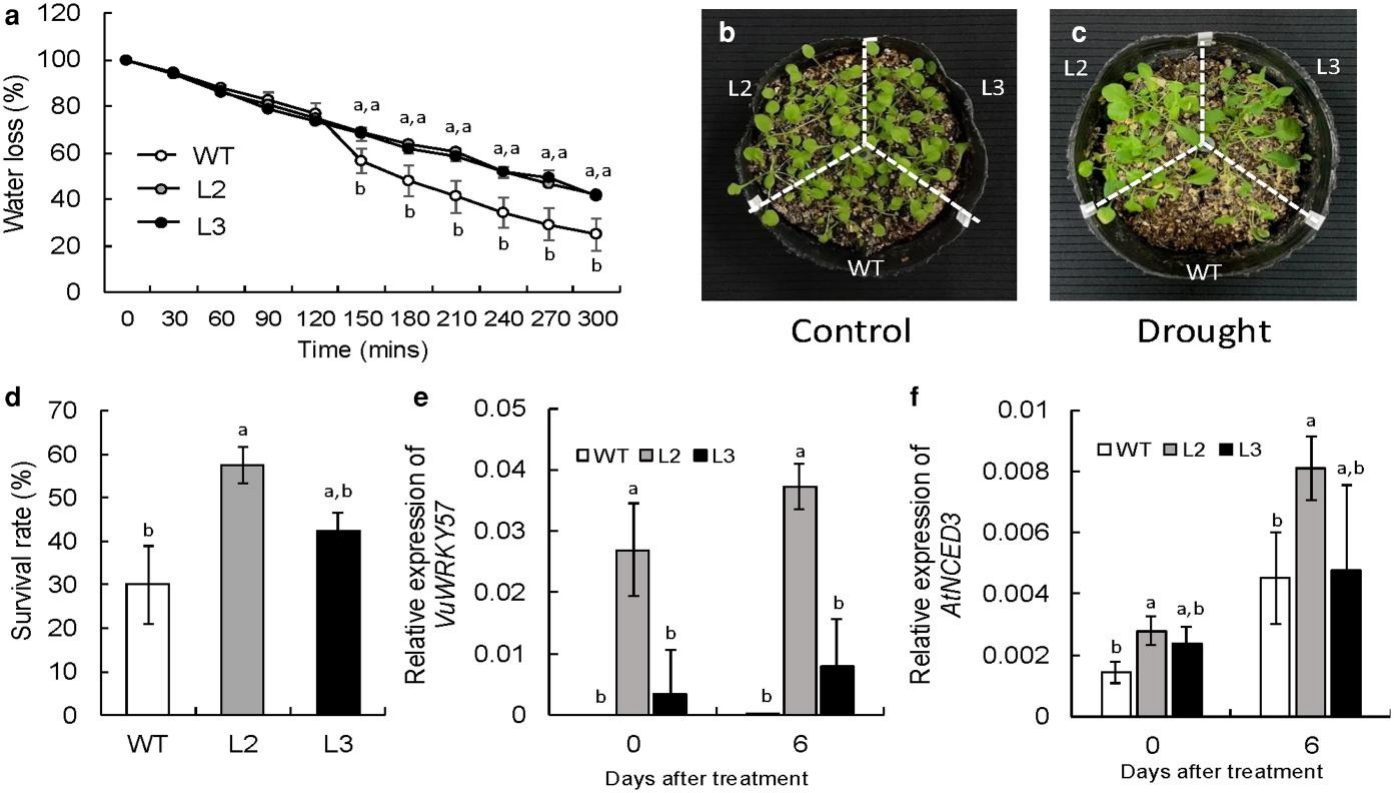
Drought stress tolerance in wild type and *VuWRKY57*-overexpression lines (L2, L3) in *Arabidopsis*. (a) Leaf water loss using detached leaves. (b, c) Plant appearance. (d) Survival rate after 35 days stopped irrigation. (e) *VuWRKY57* expression. (f) *AtNCED3* expression. White, grey, and black circles and bars mean wild type, line2 (L2), and line3 (L3), respectively. Means followed by different letters differ significantly (*P* < .05, Tukey’s test).

*VuWRKY57*-overexpression *Arabidopsis* plants had significantly better drought tolerance than the wild type (Fig. 6b and c), significantly better water retention, survival rate, and higher leaf temperature (Fig. 6a, d and Supplementary Figure S2). Compared with the wild-type plants, the overexpression plants, especially line2, showed an increase of *VuWRKY57* and *AtNCED3* expressions (Fig. 6e and f). Although *AtWRKY57 expression* of wild type and the overexpression lines increased by drought stress, the expression of overexpression lines under drought stress was lower than that of wild type (Supplementary Figure S4). These results strongly support our hypothesis that drought stress tolerance of cowpea is due to activated WRKY expression.

## Discussion

### Rapidly closure of stomata by abscisic acid biosynthesis through the VuWRKY57 of cowpea under drought stress

In order to thrive under drought stress, plants use sophisticated responses and adaptations, including stomatal closure to prevent water loss, accumulation of osmolytes, and ROS scavenging (Joshi et al. 2016, Fahad et al. 2017, Ullah et al. 2018). Here, we demonstrated that WRKY performs a crucial function in the drought tolerance of cowpea compared with soybean. ABA is a key hormone in the mechanism of cowpea drought tolerance. The quickly accumulated ABA content is a typical plants’ response to water deficit and leads to stomatal closure and the expression of numerous stress-responsive genes (Yoshida et al. 2015, Agurla et al. 2018). Cowpea responds immediately to drought stress by expressing *VuNCED* genes, which promote the biosynthesis of ABA, which induces rapid stomatal closure (Iuchi et al. 2000). Cowpea expressed *VuNCED1* in roots and increased ABA content sooner under drought stress than soybean (Fig. 2a c, d and f). Consequently, rapid closure of stomata maintained an RWC of leaf and stem, which conferred drought tolerance (Fig. 1). These results suggest that the rapid expression of *VuNCED1* is a key factor in the drought tolerance of cowpea.

WRKY transcription factors regulate many biotic and abiotic stress responses (Jiang et al. 2014; Chen et al. 2020). WRKY family proteins are implicated in the regulation of genes involved in drought stress responses (Gao et al. 2018). In *Arabidopsis*, activated expression of *WRKY57* improved drought tolerance by elevating ABA levels via increased *AtNCED3* expression (Jiang et al. 2012). In this study, drought stress induced the expression of *VuWRKY57* and *GmWRKY32*, *AtWRKY57* homologues, in parallel with that of *NCED* genes (Figs 2 and 3). In particular, the expression of *VuWRKY57* in cowpea roots rapidly increased after the imposition of drought stress. VuWRKY57 was localized in the nucleus and interacted with the W-box of the *VuNCED1* promoter (Fig. 4). These results suggest that VuWRKY57 plays a key role in drought tolerance of cowpea by promoting rapid ABA biosynthesis via direct binding to the *VuNCED1* promoter.

### Overexpression of *VuWRKY57* in *Arabidopsis* and implication for crop improvement

In *Arabidopsis*, *AtWRKY57* overexpression under drought stress reduced leaf water loss, increased ABA content via induction of *AtNCED3*, and consequently conferred drought tolerance (Jiang et al. 2012). In rice, however, *AtWRKY57* overexpression under drought stress did not significantly change the relative transcript levels of *OsNCED5* or ABA content (Jiang et al. 2016). Although GhWRKY1-like from *Gossypium hirsutum* does not directly bind to the promoter region of *AtNCED3,* which plays a major role in the regulation of ABA synthesis in response to water deficit, GhWRKY1-like may indirectly manipulate *AtNCED3*-mediated drought responses via directly promoting the transcriptional levels of *AtNCED5*, *AtNCED6,* and *AtNCED9* (Hu et al. 2021). Therefore, there may be different regulatory mechanisms between transgenic *Arabidopsis* and transgenic rice.

*VuWRKY57*-overexpressing *Arabidopsis* plants had high survival rates under drought stress and increased expression of *AtNCED3* (Fig. 5). In addition, the expression of *AtWRKY57* under drought stress was lower in overexpression plants compared with wild type (Supplementary Figure S4), and they had a higher leaf temperature during early drought stress than the wild type (Supplementary Figure S2), perhaps due to stomatal closure (Reynolds-Henne et al. 2010). From the transgenic plants analysis, it was indicated that VuWRKY57 could alleviate drought stress in *Arabidopsis*. In addition, the expression of *GmWRKY32* and *VuWRKY57* was positively correlated with the time of survival under drought stress (Supplementary Figure S3). These results suggested that the activation of *GmWRKY32* and *VuWRKY57* expression confers drought tolerance in soybean and cowpea. Additionally, AtWRKY57 is involved not only in ABA accumulation, but also in ABA response, and positively regulates hyperosmotic stress responses in *Arabidopsis* and rice (Jiang et al. 2012, 2016). Cowpea is also salinity tolerant (Win and Oo 2015). Establishment of the functions of VuWRKY57 will enable the improvement of osmotic tolerance such as drought stress and salinity stress, although further analysis is needed.

## Conclusion

This study highlights the pivotal role of VuWRKY57 in the drought-tolerance mechanism of cowpea. Through its direct interaction with the *VuNCED1* promoter, VuWRKY57 facilitates rapid ABA biosynthesis, which triggers stomatal closure and conserves water during drought stress. Additionally, the overexpression of *VuWRKY57* in *Arabidopsis* further confirms its contribution to enhanced drought tolerance, demonstrating increased survival rates and improved water retention under stress conditions. These findings not only provide a deeper understanding of the genetic and molecular mechanisms underpinning drought tolerance in cowpea but also suggest potential strategies for improving drought resilience in other crops. Future research could explore the interaction of VuWRKY57 with other transcription factors and its broader role in abiotic stress responses, including salinity tolerance. This knowledge could guide the development of genetically engineered crops capable of thriving under increasingly challenging environmental conditions.

## Supplementary Material

plaf038_Supplementary_Data

## Data Availability

All data supporting the findings of this study are available within the paper and supplementary data published online.
