# Supplementary material for: Drought tolerance of cowpea is associated with rapid abscisic acid biosynthesis via VuWRKY57 in root
Source: AoB Plants. 2025 Jul 8;17(4):plaf038. doi: 10.1093/aobpla/plaf038 (PMC12280871; doi:10.1093/aobpla/plaf038)
Supplement: plaf038_Supplementary_Data [file plaf038_supplementary_data.pptx]

## Slide 1
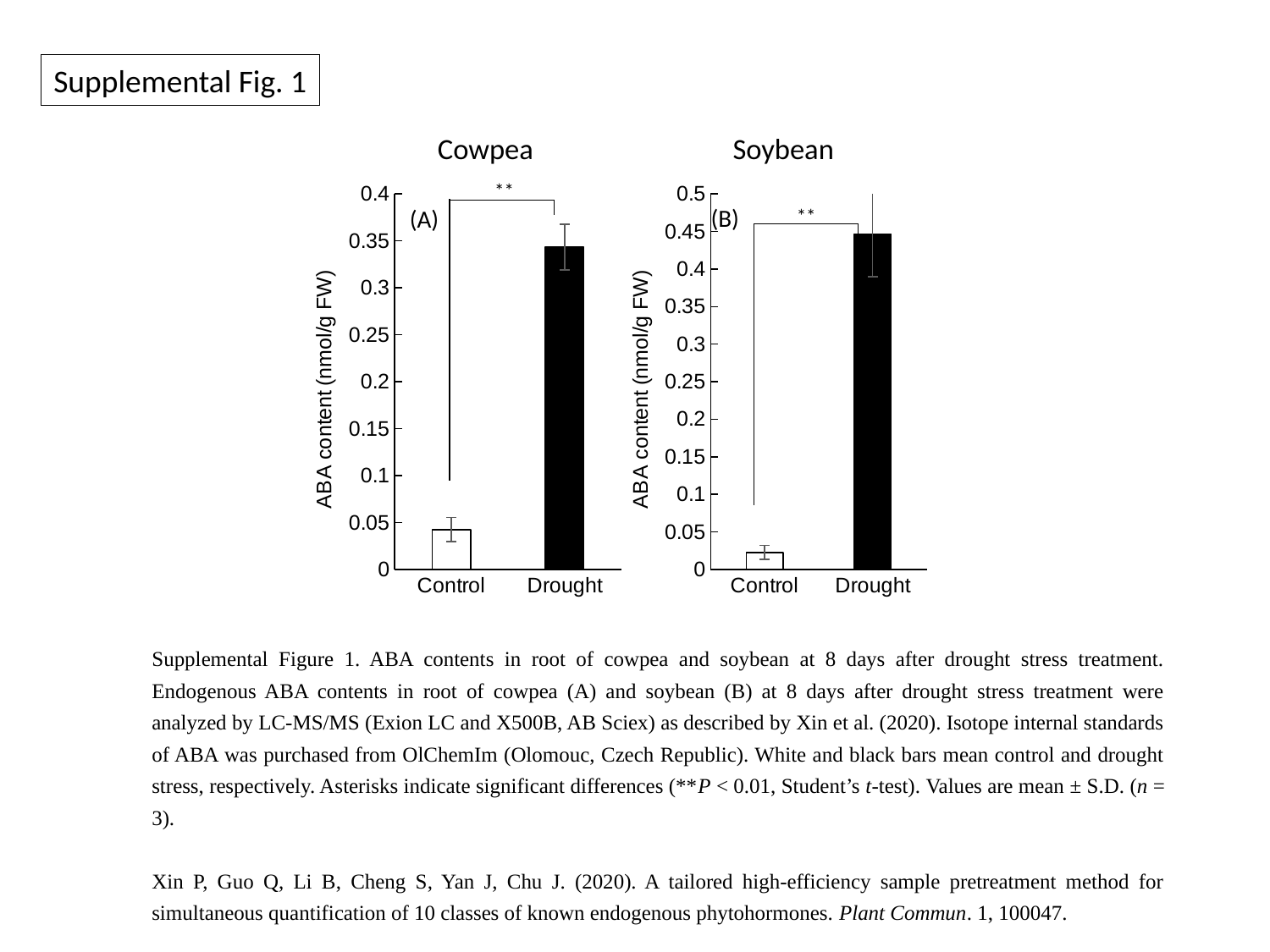

Supplemental Fig. 1
Cowpea
Soybean
**
### Chart
| Category | Root |
|---|---|
| Control | 0.04248253908450394 |
| Drought | 0.34337859858177505 |
### Chart
| Category | Root |
|---|---|
| Control | 0.02244838351687541 |
| Drought | 0.4467085386513947 |(B)
(A)
**
Supplemental Figure 1. ABA contents in root of cowpea and soybean at 8 days after drought stress treatment. Endogenous ABA contents in root of cowpea (A) and soybean (B) at 8 days after drought stress treatment were analyzed by LC-MS/MS (Exion LC and X500B, AB Sciex) as described by Xin et al. (2020). Isotope internal standards of ABA was purchased from OlChemIm (Olomouc, Czech Republic). White and black bars mean control and drought stress, respectively. Asterisks indicate significant differences (**P < 0.01, Student’s t-test). Values are mean ± S.D. (n = 3).
Xin P, Guo Q, Li B, Cheng S, Yan J, Chu J. (2020). A tailored high-efficiency sample pretreatment method for simultaneous quantification of 10 classes of known endogenous phytohormones. Plant Commun. 1, 100047.

## Slide 2
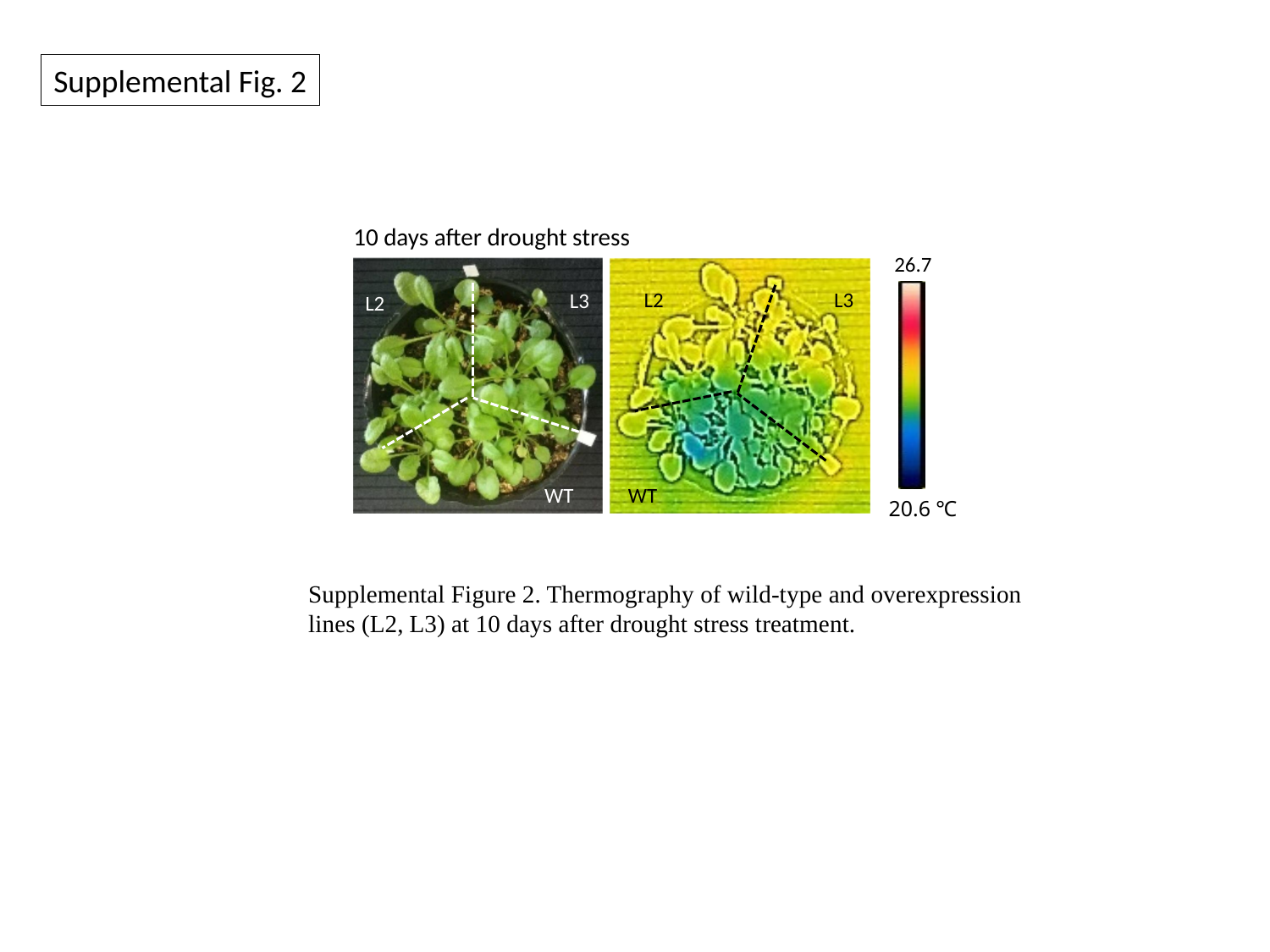

Supplemental Fig. 2
10 days after drought stress
26.7
L3
L2
L3
L2
WT
WT
20.6 ℃
Supplemental Figure 2. Thermography of wild-type and overexpression lines (L2, L3) at 10 days after drought stress treatment.

## Slide 3
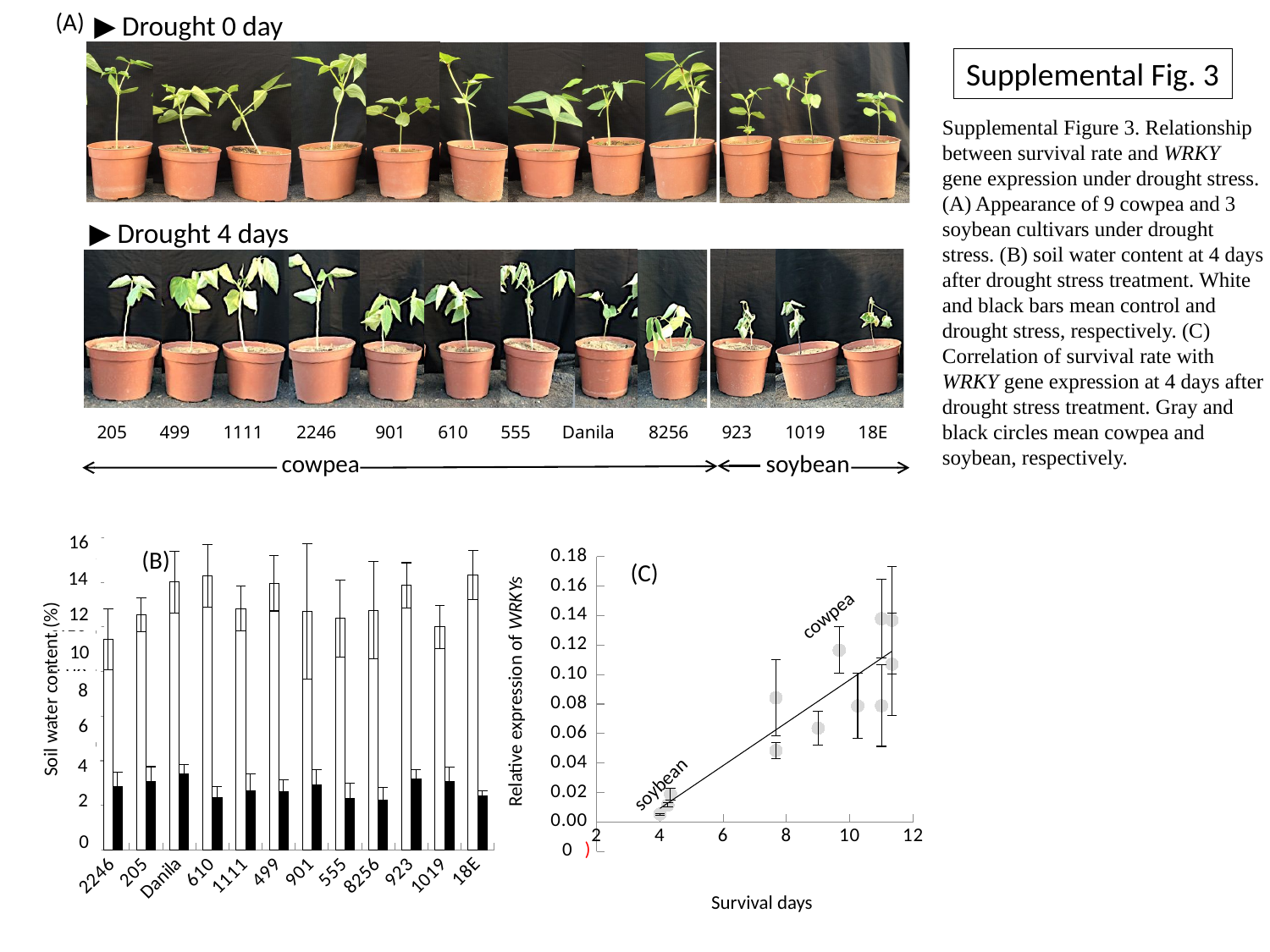

(A)
▶ Drought 0 day
Supplemental Fig. 3
Supplemental Figure 3. Relationship between survival rate and WRKY gene expression under drought stress. (A) Appearance of 9 cowpea and 3 soybean cultivars under drought stress. (B) soil water content at 4 days after drought stress treatment. White and black bars mean control and drought stress, respectively. (C) Correlation of survival rate with WRKY gene expression at 4 days after drought stress treatment. Gray and black circles mean cowpea and soybean, respectively.
▶ Drought 4 days
205
499
1111
2246
901
610
555
Danila
8256
923
1019
18E
cowpea
soybean
### Chart
| Category | Control | Drought |
|---|---|---|
| 2246 | 0.09436347548924065 | 0.028364743445239073 |
| 205 | 0.1054863801502876 | 0.030810010964760784 |
| Danila | 0.12001476014086228 | 0.03404543141876738 |
| 610 | 0.12285904116560152 | 0.02347933042692804 |
| 1111 | 0.10813499861133802 | 0.026448400083033223 |
| 499 | 0.11949100758415813 | 0.026272359723199357 |
| 901 | 0.1068292201661132 | 0.029241480413730495 |
| 555 | 0.10377604715637755 | 0.023024077596313454 |
| 8256 | 0.10750117333030024 | 0.0223451576706752 |
| 923 | 0.11861696087474902 | 0.03185916757878824 |
| 1019 | 0.09999153238129524 | 0.03074267377173663 |
| 18E | 0.12309241392204925 | 0.024442505969917455 |16
(B)
### Chart
| Category | |
|---|---|(C)
14
12
cowpea
10
Soil water content (%)
8
Relative expression of WRKYs
6
4
soybean
2
0
0
0
 Survival days

## Slide 4
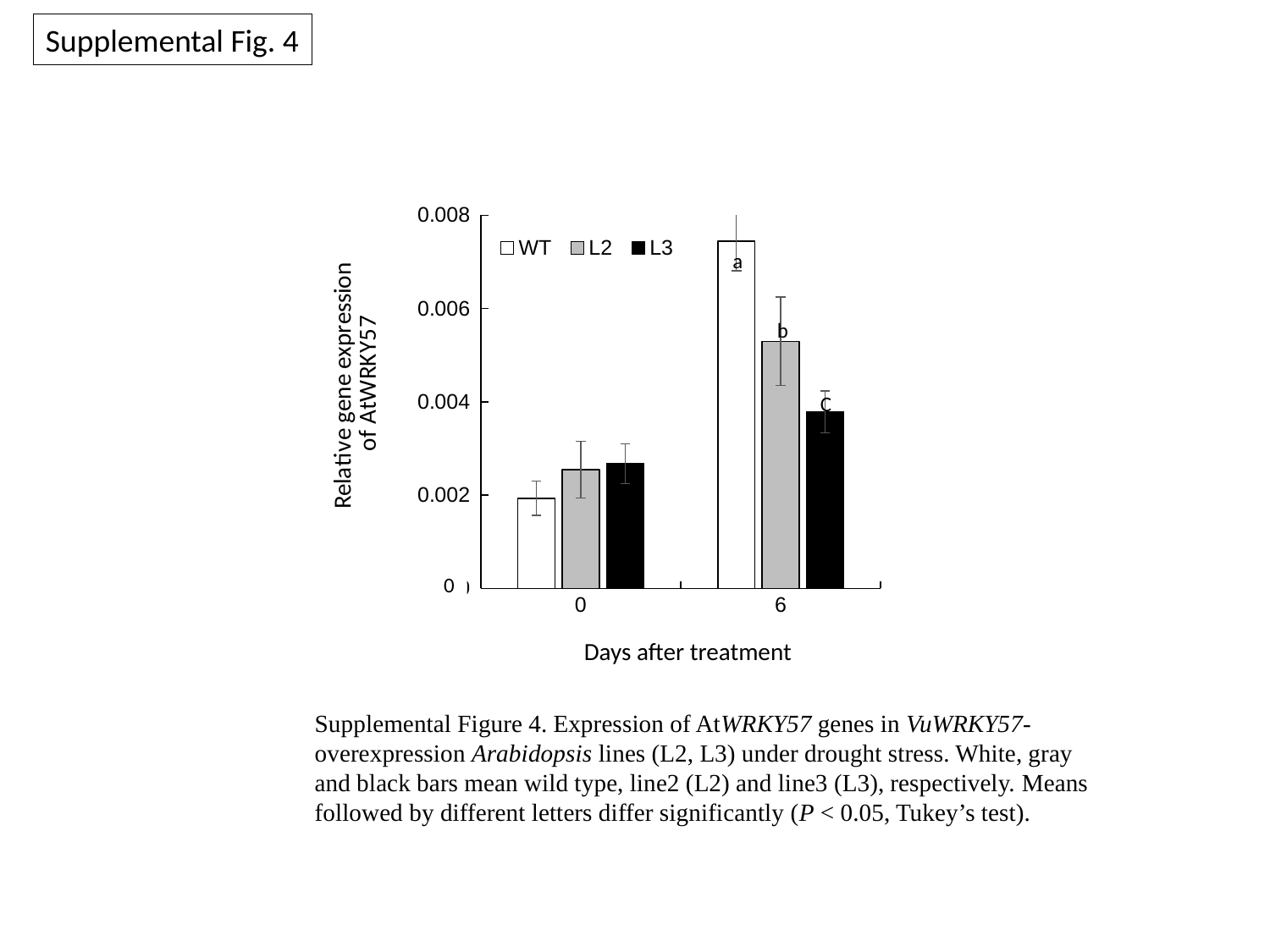

Supplemental Fig. 4
### Chart
| Category | WT | L2 | L3 |
|---|---|---|---|
| 0 | 0.0019331492547252616 | 0.002550250105023253 | 0.0026735390859386706 |
| 6 | 0.0074457543663269095 | 0.005295524315227899 | 0.003786331000950198 |a
b
C
0
Days after treatment
Supplemental Figure 4. Expression of AtWRKY57 genes in VuWRKY57-overexpression Arabidopsis lines (L2, L3) under drought stress. White, gray and black bars mean wild type, line2 (L2) and line3 (L3), respectively. Means followed by different letters differ significantly (P < 0.05, Tukey’s test).
